# The SNAP trial: a double blind multi-center randomized controlled trial of a silicon nitride versus a PEEK cage in transforaminal lumbar interbody fusion in patients with symptomatic degenerative lumbar disc disorders: study protocol

**DOI:** 10.1186/1471-2474-15-57

**Published:** 2014-02-25

**Authors:** Roel FMR Kersten, Steven M van Gaalen, Mark P Arts, Kit CB Roes, Arthur de Gast, Terry P Corbin, F Cumhur Öner

**Affiliations:** 1 Clinical Orthopaedic Research Center–midden Nederland (CORC-mN), Department of Orthopaedics, Diakonessenhuis, Utrecht/Zeist, The Netherlands; 2 Department of Neurosurgery, Medical Center Haaglanden, the Hague, The Netherlands; 3 Julius Centre for Health Sciences and Primary Care, University Medical Center Utrecht, Utrecht University, Utrecht, The Netherlands; 4 Corbin & Company, Maple Grove, MN, USA; 5 Department of Orthopaedics, University Medical Center Utrecht, Utrecht University, Utrecht, The Netherlands

**Keywords:** Disc degeneration, Spondylolisthesis, Lumbar interbody fusion, PEEK, Silicon nitride, Ceramic implant, Randomized controlled trial

## Abstract

**Background:**

Polyetheretherketone (PEEK) cages have been widely used in the treatment of lumbar degenerative disc disorders, and show good clinical results. Still, complications such as subsidence and migration of the cage are frequently seen. A lack of osteointegration and fibrous tissues surrounding PEEK cages are held responsible. Ceramic implants made of silicon nitride show better biocompatible and osteoconductive qualities, and therefore are expected to lower complication rates and allow for better fusion.

Purpose of this study is to show that fusion with the silicon nitride cage produces non-inferior results in outcome of the Roland Morris Disability Questionnaire at all follow-up time points as compared to the same procedure with PEEK cages.

**Methods/Design:**

This study is designed as a double blind multi-center randomized controlled trial with repeated measures analysis. 100 patients (18–75 years) presenting with symptomatic lumbar degenerative disorders unresponsive to at least 6 months of conservative treatment are included. Patients will be randomly assigned to a PEEK cage or a silicon nitride cage, and will undergo a transforaminal lumbar interbody fusion with pedicle screw fixation. Primary outcome measure is the functional improvement measured by the Roland Morris Disability Questionnaire. Secondary outcome parameters are the VAS leg, VAS back, SF-36, Likert scale, neurological outcome and radiographic assessment of fusion. After 1 year the fusion rate will be measured by radiograms and CT. Follow-up will be continued for 2 years. Patients and clinical observers who will perform the follow-up visits will be blinded for type of cage used during follow-up. Analyses of radiograms and CT will be performed independently by two experienced radiologists.

**Discussion:**

In this study a PEEK cage will be compared with a silicon nitride cage in the treatment of symptomatic degenerative lumbar disc disorders. To our knowledge, this is the first randomized controlled trial in which the silicon nitride cage is compared with the PEEK cage in patients with symptomatic degenerative lumbar disc disorders.

**Trial registration:**

NCT01557829

## Background

Chronic low back pain is an important reason for patients to visit general practitioners. In Europe, estimates of lifetime prevalence of chronic low back pain range from approximately 60–90% [1]. It is one of the leading causes of activity limitation in adults and results in high socio-economic costs and loss of quality of life [2]. The exact cause of chronic low back pain is often unknown, but degenerative disorders of the intervertebral disc are held responsible [3]. The pain can be eliminated by stabilizing the degenerative segment, for example as seen in the successful treatment of degenerative joints with an arthrodesis [4,5]. Spinal fusion is commonly used for stabilizing degenerative and isthmic spondylolisthesis and severe, painful disc degeneration.

In a spinal fusion, two or more vertebrae are fused after a bone bridge is created between the vertebrae, either posterior, interbody or both. Originally, bone grafts were used to promote interbody fusion. However, several complications were associated with the use of grafts. These include donor site morbidity, a decrease in the intervertebral disc space height due to graft collapse, graft subsidence, graft retropulsion, graft resorption, fusion failure with subsequent pseudarthrosis and prolonged healing time [6,7].

As an alternative for bone grafts, interbody cages were developed [7]. They are designed to be filled with bone, allowing bony fusion through the cage to the adjacent vertebrae. Both material and design of the cage play an important role in correcting spinal deformities and creating an optimal environment for spinal fusion [8-10]. The development of a solid bony fusion is influenced by mechanical and biological factors. For example, the size and geometry of the cage determines the initial mechanical stability [11,12]. Furthermore cage stiffness is an important factor in stress shielding [13,14]. Biological factors, such as the osteointegration of the surface of the cage, influence quality, speed and attachment of newly formed bone [8].

Cages allow for direct axial load bearing and restore of height of the intervertebral and foraminal space. Initially, interbody cages were implanted in pairs via the traditional posterior lumbar interbody fusion (PLIF) technique. More recently, a larger single oblique cage is used that provides more stability [15,16].

Polyetheretherketone (PEEK) materials were used in aerospace and aviation industries before researchers began exploring them in medical devices, mainly in trauma and femoral components of hip prosthesis [17,18]. Besides being radiolucent, PEEK is relatively inert and does not provoke a strong foreign body reaction in vivo [19]. During the late 1990’s the first PEEK cages for spinal fusion became available. High fusion rates and good to excellent clinical outcomes have been reported compared to titanium cages and bone grafts [20,21]. Most spine surgeons therefore prefer PEEK cages over other cages.

To allow some visualization on radiograms and CT, radio-opaque markers are present in PEEK cages. The major advantage of PEEK cages over the metal cages is that they produce less artifacts on CT or MR scans. However, a radiolucent cage could also contribute to the difficulty of radiographic assessment of its exact position in the spine. For example, placement of the cage during surgery is less accurate, and follow-up imaging is more difficult. This is important to determine the cause of ongoing symptoms and/or to determine if fusion has occurred. Additional problems observed include a 14.3% rate of subsidence in patients with PEEK cages after lumbar interbody fusion [22]. Furthermore, posterior migration of a component of a PEEK cage has been reported [23]. It has also been reported that PEEK cages are generally encapsulated by a thin fibrous tissue layer rather than bone growing in intimate contact with the polymer [24].

Better osteointegration of the cage is believed to minimize the rate of subsidence and migration. Therefore, researchers have been working on materials that mimic the mineral content of bone for many years [25]. Ceramic implants can be manufactured with a rough surface and have the potential to show a better integration with the host bone, which facilitates the attachment of bone to the implant rather than the fibrous encapsulation [24]. Ceramics are strong and light-weight and have desirable imaging properties, free from artifacts on CT and MRI [26].

Silicon nitride (Si_3_N_4_) is a ceramic with a compression strength exceeding the usual plastic and metal materials used for interbody cages. Unlike many other ceramics, silicon nitride resists brittle fractures; its toughness exceeds that of alumina, a material with 30+ years of use in joint replacements [27]. Silicon nitride is also highly compatible with standard imaging techniques. The material is free from artifacts on radiogram, CT and MRI images [26]. Several studies have demonstrated its biocompatibility and its mechanical and osteoconductive qualities in vitro [28-32]. Furthermore, compared to PEEK and titanium, silicon nitride has a decreased bacterial activity on its surface [33,34]. Based on good results in vitro, silicon nitride is used in the development of bearings that can improve wear and longevity of knee and hip prosthesis [32].

A preliminary study with silicon nitride interbody cages showed good clinical and radiological results in 2 patients 1 year after a transforaminal lumbar interbody fusion procedure [35]. Sorrell et al. presented the results of a 10 year clinical follow-up study [36]. In this study 30 patients underwent anterior interbody fusion of the lumbar spine using silicon nitride cages. They found a durable interbody fusion after 5 years (21 out of 22 patients) and after 10 years (16 out of 16 patients). Please note there was a 47% loss of follow-up. Silicon nitride materials received the CE Mark and FDA market clearance for its use as interbody cages in 2008. They have been used in the US for over 3 years, with no adverse events reported [32].

Compared to PEEK cages silicon nitride cages are expected to have lower complications rates and allow higher fusion rates due to better biocompatible and osteoconductive qualities. The purpose of this study is to compare the clinical outcomes and fusion rates of PEEK cages with silicon nitride cages in patients with symptomatic degenerative lumbar disc disorders.

## Methods/Design

In our study, PEEK and silicon nitride interbody cages will be compared in the treatment of degenerative lumbar disc disorders. This non-inferiority study is designed as a multi-center (two center) clinical observer and patient blind randomized controlled trial with 2 parallel treatment groups. The multi-center design is needed in order to collect enough patients for reasons of statistical power. To minimize observer bias, both patients and clinical observers will be blinded for treatment during follow-up. Clinical observers will not analyze radiograms and CT because the silicon nitride cages are clearly visible. The follow-up is 2 years, in which patients will fill out several questionnaires and are examined both clinically and radiologically.

### Patient selection

Participation in our study will be requested from patients (18–75 years old) who visit the outpatient clinic in one of the participating hospitals. Patients must present with a history of chronic low back pain with or without leg pain that did not respond to conservative treatment and disc degeneration of Pfirrmann Grade III [37] or higher and/or isthmic or degenerative spondylolisthesis of Grade I or II, confirmed by MRI.

The treating physician will discuss this study with the patient and if the patient fulfills all inclusion criteria (Table 1), the information form and informed consent form is handed out to the patient. The patient can read subsequently at leisure at home.

**Table 1:** Inclusion and exclusion criteria

*Inclusion criteria*	• Male and female patients age 18–75 years
	• Chronic low back pain unresponsive to at least six months of conservative care
	• MRI and standing x-ray evidence of Pfirrmann Grade III or greater disc
	• Degeneration and/or degenerative or isthmic spondylolisthesis of Grade I or II
	• Signed informed consent
*Exclusion criteria*	• Osteoporosis
	• Patients with prior failed fusion at the same level
	• Degenerative scoliosis
	• Degenerative spondylolisthesis greater than Grade II
	• Pregnancy
	• Psychiatric or mental disease
	• Alcoholism (drinking more than 5 units per day)
	• Active infection or prior infection at the surgical site
	• Active cancer
	• Insufficient language skills to complete questionnaires
	• Participation in another study
	• More than two symptomatic levels that need fusion
	• Planned (e)migration abroad in the year after inclusion

Patients who decide to participate in our study are scheduled for an appointment with the researcher at the outpatient clinic of the hospital. During this visit, the patient is extensively informed about the backgrounds, the objectives, the investigational design and the assessments of the investigation and the possible advantages and disadvantages of the investigation. All this information provided by the researcher matches the earlier provided patient information form. The patient is requested to sign the informed consent. Pre-operative baseline data will then be collected for the outcome scores as well as patient’s demography. A neurological examination is performed, the MRI and other tests are reviewed and the surgery is discussed. All patients preoperatively visit an anesthesiologist for standard medical assessment. All patients will be operated under general anesthesia.

### Randomization

Patients who meet the inclusion and exclusion criteria, and have given informed consent are allocated the next available investigational number (Patient ID number) and will be randomly allocated to one of two groups (treatment A or treatment B) by use of a centralized 24-hour computerized randomization system that allows internet randomization (Sealed Envelope Ltd. London). After completing follow-up at 2 years post-surgery both patient and researcher will be informed which cage was used.

### Surgical management

Patients will undergo a transforaminal lumbar interbody fusion with an oblique single PEEK or SiN cage (Amedica Corporation, Salt lake City, Utah) supplemented by pedicle screw fixation, as described by Harms et al. [38]. Design of the PEEK cage is similar to the SiN cage. Autograft bone extracted from locally excised bone from the lumbar spine will be used for cage filling. After surgery, patients will be admitted for 3–4 days. Patients are encouraged to mobilize as soon as possible. A lumbar support orthosis is not prescribed.

### Outcome measurements

Several validated questionnaires described below will be used for outcome assessments. During intake, a basic physical exam with neurological examination (muscle strength, reflexes) and additional assessments as required per normal practice will be performed to ensure that the patient can undergo surgery safely. During follow-up visits at 3, 6, 12 and 24 months the neurological examination will be repeated. See Table 2 for the patient follow-up chart.

**Table 2:** Follow-up chart

	**Intake**	**Admission**	**3 months**	**6 months**	**12 months**	**24 months**
Visit	1	2	3	4	5	6
Demography	X					
Study information + informed consent	X					
Randomization	X					
Surgery		X				
Operative data		X				
Neurological examination	X		X	X	X	X
Questionairres:	X		X	X	X	X
- Roland Morris disability questionairre						
- SF-36						
- VAS back						
- VAS leg						
- Working status						
Likert scale		X	X	X	X	X
X-rays	X	X	X	X	X	X
CT					X	
MRI	X					
Complications		X	X	X	X	X

#### Primary outcome measure

Primary outcome will be measured by the Roland Morris Disability Questionnaire (RMDQ). The 24 point RMDQ is a widely used patient-completed measure of health outcome for low back pain [39-41]. The patient will complete the Dutch version of the questionnaire, which is validated for the Dutch population [42], and the sum of the scores will be used to measure disability. The score ranges from 0 to 24, with a higher score indicating more severe disability. Primary objective is to measure the average improvement in RMDQ for the silicon nitride patients versus those that receive similar-shaped PEEK cages.

#### Secondary outcome measures

*SF-36:* The SF-36 will be used as the generic quality of life questionnaire [43,44]. The SF-36 questionnaire has been applied and validated numerous times for intervention studies with back pain and spine surgery. The questionnaire relates to the analysis of the general functional status of patients. The questions are divided in eight domains:

● Physical functioning

● Physical role limitations

● Emotional role limitations

● Social functioning

● Physical pain

● General mental health

● Vitality

● General health perception

Each domain is converted to a 0 to 100 score, a higher score indicating a better health condition. The eight domains are also combined into a physical and psychological summary score. These are converted to range from 0 to 100 with an average person at 50 and a standard deviation of 10 points.

*Pain (Back and Leg VAS):* The pain intensity in the back and legs are rated by the patient on a 100 mm horizontal visual analog scale (VAS). The two ends of the scale are “no pain” at 0 mm and “the most terrible pain I can imagine” at 100 mm. The patient is asked to mark the scale based on the average pain intensity during the week prior to the visit to the outpatient clinic. During each visit, the patient will complete one VAS for the pain in either leg, and one VAS for back pain.

*Likert score:* Recovery is rated by the patient on a 7-point Likert score in which 1 defines complete recovery and 7 is worse than ever. Likert score will be dichotomized in good recovery (‘complete recovery’ and ‘almost complete recovery’) and bad recovery (‘little recovery’ to ‘worse than ever’). Patient will complete the Likert score at the day of discharge from the hospital and during each follow-up visit.

*Radiographic Images (Plane radiogram, MRI, CT):* A pre-operative MR and a set of standing plane radiograms of the lumbar spine will be collected for all patients. Pre-operative disc degeneration will be evaluated on the MR scan by the method of Pfirrmann [37]. Patient fusion status will be evaluated according to the criteria mentioned by Burkus et al. which are based on qualitative observations [45,46]. Determination of fusion involves the radiographic evaluation of angular changes in spinal alignment, assessment of the device-host interface, and identification of new bone formation and bone remodeling [46]. Anterior – posterior radiograms will be collected after 3, 6, 12 and 24 month. After one year, a CT scan (Siemens sensation 16, 3.0 mm slice) of the lumbar spine will be collected to monitor new bone formation and bone remodeling within and around the central core of the cages. Two radiologists will independently analyze the lumbar radiograms and CT. Disagreement between the radiologists will be resolved by consensus.

### Complications, adverse events, additional surgery

The investigators will record all complications and adverse events accurately. These will be grouped in the following categories:

● Infections, grouped as superficial wound infections and deep wound infections

● Post-surgical hematoma

● Increased neurological symptoms

● Venous thrombosis

● Other (serious) adverse events

All adverse events and complications will be monitored and followed up until stable or resolved during the course of the study. Each adverse event will be reported to the operating surgeon and will be associated to the type of cage used to qualify the event to be related. Code breaking will occur by the clinical observer or operating surgeon if the clinical condition of the patient necessitates this*.* Early termination of the study will be decided if necessary.

#### Additional surgery

All additional surgeries during the follow-up period that are related to surgery will be recorded. Any additional spine surgery at the operated level will be considered as a complication and a poor result.

### Withdrawal of participants from the trial

A participant may be withdrawn from the clinical study for the following reasons:

● Patients may choose to withdraw from the study under the terms of the Declaration of Helsinki and their consent documentation without having to give a reason

● Any unanticipated adverse reaction which is, in the opinion of the researcher, related to the treatment and will endanger the well-being of the patient if treatment is continued

● The development of any intercurrent illness(es), infection or condition(s) that might interfere with the clinical investigation

● Non-compliance with the study procedures deemed by the investigator to be sufficient to cause discontinuation

● Any problem deemed by the Investigator to be sufficient to cause discontinuation

All patients discontinued from the investigation due to an unanticipated adverse reaction, directly related to the investigation, will be treated until the reaction resolves. The researcher will clearly document the date and reason(s) for the patient withdrawal. Patients who have withdrawn from the study will not be replaced if they have received investigation treatment. If possible, any procedures or assessments planned for the patient on withdrawal from the investigation should be performed when intention to withdraw the patient is announced. Patients who are withdrawn prior to receiving treatment will be replaced.

### Data management

All data recorded during intake, hospitalization and follow-up visits will be de-identified. Participants will be identified by a unique investigational number (Patient ID number) allocated during intake. Primary and secondary outcome variables, information gathered during intake and hospitalization and all complications, additional surgery, adverse events and withdrawals will be entered by the researcher into an electronic data capture system (Acumen Healthcare Solutions, LLC, Plymouth, Minnesota, USA). The source documents will be stored in the hospital where the patient underwent the surgical procedure and shall be retained for a period of minimal 5 years after the study completion or longer if deemed necessary.

### Statistical considerations

#### Sample size

The sample size calculation is based on the primary objective to compare the silicon nitride and PEEK cages with respect to improvement in RMDQ score and to demonstrate that the silicon nitride cage is non-inferior to the PEEK cage.

In a large spinal fusion cohort study, Robertson [47] found a mean RMDQ improvement of about 10 points. Scheufler also noted an improvement from a pre-treatment score of 17 to 7 at eight months post-op, with a standard deviation of 4 [48]. Both studies included patients with back pain from degenerative disc disease and degenerative spondylolisthesis.

The maximal difference between the treatment arms that could be considered potentially no longer clinically relevant for the RMDQ is thus a difference in improvement of 2–3.5 points [39,41,49]. We therefore consider a non-inferiority margin of 2.6 points between the treatment arms to reflect the maximal difference that is not clinically relevant. Non-inferiority is to be demonstrated based on a one-sided confidence interval with significance level of 2.5% for the difference between the two treatment arms. Assuming a standard deviation of 4 points, 50 patients per arm provide 90% power to demonstrate non-inferiority within a non-inferiority margin of 2.6 points. The total of 100 patients shall be randomized into two groups to minimize bias. This sample size is based on comparing treatment groups with a *t*-test. The actual analysis is a repeated measurements analysis with baseline as covariate, which is more efficient (requiring less patients, at least about 10% if the correlation between baseline and endpoint is 0.3). Thus, no additional sample size increase is incorporated to account for drop-out. Sensitivity analyses to assess impact of drop outs will be performed.

#### Statistical analysis

The primary analysis will be on the change from baseline in RMDQ score. This will be analyzed based on a mixed model for repeated measurements, including baseline RMDQ as covariate and treatment and center as factors. No imputation will be applied for this analysis. The primary comparison will be at 12 months of follow-up.

Sensitivity analyses to assess impact of drop outs will be performed. These will include an analysis based on Last Observation Carried Forward imputation, as well as multiple imputations based on differential patterns of drop out/missing data reasons.

An exploratory analysis of the distribution of the individual improvements in change from baseline in RMDQ score versus the fusion rate (in three categories) within treatment groups will be performed to assess the extent to which both are consistent.

Other continuous outcomes assessed at each visit will be analyzed similarly. Dichotomous outcomes will be compared between treatment groups based on Z-tests for comparing proportions, with results expressed as 95% confidence intervals for the difference in proportions.

### Ethical considerations

This study is designed in concordance with the declaration of Helsinki. The protocol has been reviewed and approved by the local medical ethical committee (Verenigde Commissies Mensgebonden Onderzoek). The general board of the participating hospitals also agreed with the protocol. Informed consent will be obtained before participation in this study. Patients are informed they are free to refuse participation. If they choose to participate they may withdraw from this study at any time without comprising further medical care. No financial rewards will be present for patients who agree to participate.

## Discussion

PEEK cages are widely used in the treatment of lumbar degenerative disc disorders, and show good clinical results [20,21]. Nevertheless, complications such as subsidence and migration of the cage are frequently seen [22,23]. A lack of osteointegration and fibrous tissues encapsulating PEEK cages are held responsible [24]. Ceramic implants made of silicon nitride show better biocompatible and osteoconductive qualities [28-34]. Therefore it is expected that the use of silicon nitride cages decrease such complications by better fusion rates. A study design of a double blind multi-center randomized controlled trial is presented in this article, in which PEEK cages will be compared with silicon nitride cages in the treatment of symptomatic degenerative lumbar disc disorders. Primary objective is to show that treatment with the silicon nitride cage produces similar improvement in RMDQ at all follow-up times compared to the PEEK cage. Total follow-up is 2 years. To our knowledge, this is the first randomized controlled trial in which the silicon nitride cage is compared with the PEEK cage in patients with symptomatic degenerative lumbar disc disorders.

## Competing interests

The CORC-mN foundation of the Diakonessenhuis, the Neurosurgical Scientific Partnership The Hague of the Medical Center Haaglanden and Corbin & Company are receiving financial support for their work on this study from Amedica Corporation.

## Authors’ contributions

RK provided research design, writing and project management. SvG provided concept, research design, revision and project management. MA provided project management and revision. KR provided statistical design and analyses. AG provided revision. TC provided concept, research design, statistical design, revision and project management. FÖ provided project management and revision. All authors read and approved the final manuscript.

## Pre-publication history

The pre-publication history for this paper can be accessed here:

http://www.biomedcentral.com/1471-2474/15/57/prepub
